# Origin of myofibroblasts in liver fibrosis

**DOI:** 10.1186/1755-1536-5-S1-S17

**Published:** 2012-06-06

**Authors:** David A Brenner, Tatiana Kisseleva, David Scholten, Yong Han Paik, Keiko Iwaisako, Sayaka Inokuchi, Bernd Schnabl, Ekihiro Seki, Samuele De Minicis, Christoph Oesterreicher, Kojiro Taura

**Affiliations:** 1 University of California, San Diego, School of Medicine, San Diego, CA, USA

## Abstract

Most chronic liver diseases of all etiologies result in progressive liver fibrosis. Myofibroblasts produce the extracellular matrix, including type I collagen, which constitutes the fibrous scar in liver fibrosis. Normal liver has little type I collagen and no detectable myofibroblasts, but myofibroblasts appear early in experimental and clinical liver injury. The origin of the myofibroblast in liver fibrosis is still unresolved. The possibilities include activation of endogenous mesenchymal cells including fibroblasts and hepatic stellate cells, recruitment from the bone marrow, and transformation of epithelial or endothelial cells to myofibroblasts. In fact, the origin of myofibroblasts may be different for different types of chronic liver diseases, such as cholestatic liver disease or hepatotoxic liver disease. This review will examine our current understanding of the liver myofibroblast.

## Background

Myofibroblasts are alpha smooth muscle actin positive cells that produce extracellular matrix proteins including fibrillar collagen. Myofibroblasts are characterized immunophenotypically by a spindle or stellate shape, pale eosinophilic cytoplasm, expression of abundant pericellular matrix and fibrotic genes (vimentin, α-smooth muscle actin (α-SMA), non-muscle myosin, fibronectin, and collagen Type I) [1,2]. Ultrastructurally, myofibroblasts are defined by prominent rough endoplasmic reticulum, a Golgi apparatus producing collagen, peripheral myofilaments, fibronexus (no lamina) and gap junctions [2]. In liver fibrosis, the myofibroblasts are imbedded in the fibrous scar. In both experimental and clinical liver fibrosis, there is a close correlation between the regression of liver fibrosis and the disappearance of these myofibroblasts. There is general agreement that these myofibroblasts are the source of the excessive extracellular matrix proteins in liver fibrosis. Therefore, identifying the origin of these myofibroblasts will provide insight into the pathology of liver fibrosis and perhaps into new therapeutic targets.

**Figure 1:**
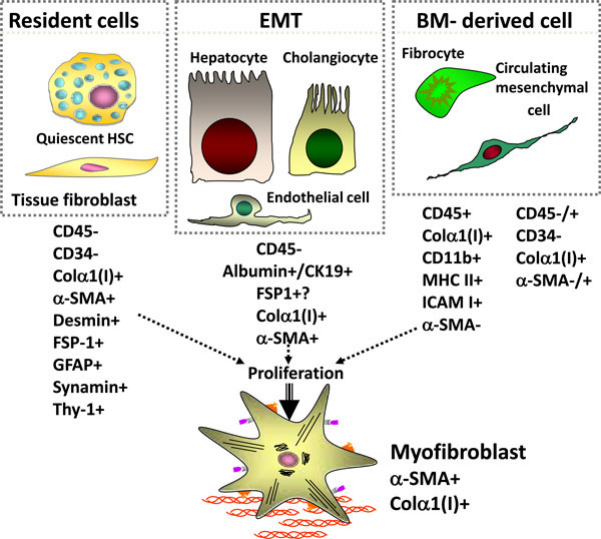
**Origin of myofibroblasts**.

There are at least three potential sources of myofibroblasts in the liver (see Figure 1). The resident mesenchymal cells, consisting of the quiescent hepatic stellate cell and the tissue fibroblasts, can potentially become myofibroblasts. These cells are characterized by CD45-, CD34-, desmin+, glial fibrillar associated protein (GFAP)+, and thy-1+. Recent studies have proposed hepatocytes, cholangiocytes, and endothelial cells can become myofibroblast through epithelial or endothelial mesenchymal transition (EMT). These cells include CD45-, albumin+ (i.e. hepatocytes), CD45-, CK19+ (i.e. cholangiocytes), or Tie2+ (endothelial cells). Finally, bone-marrow derived cells, consisting of fibrocytes and circulating mesenchymal cells, can be recruited to the injured liver to become myofibroblasts. These cells are CD45+ (fibrocytes), CD45+/- (circulating mesenchymal cells), collagen type I +, CD11d+, and MHC class II+.

The assessment of the cell fate of cells *in vivo *in mice has been greatly facilitated by the generation of transgenic mice that either express a reporter gene or express the recombinase cre under a cell-specific promoter to permanently label a cell and its progeny. We have utilized the collagen alpha1(I) GFP mouse in which the green fluorescent protein (GFP) is expressed under control of the collagen alpha1(I) promoter/enhancer [3]. These mice can then undergo chronic liver injury such as bile-duct ligation or carbon tetrachloride treatment to induce liver fibrosis and their myofibroblasts will express the GFP so are easily identified by their green fluorescence.

Our studies have assessed the potential contribution of fibrocytes to the myofibroblast population in chronic liver injury. Fibrocytes are a unique population of type I collagen expressing CD45+ cells derived from the bone marrow. Fibrocytes are defined as spindle shaped "CD45 and collagen type I (Col^+^) expressing leukocytes that mediate tissue repair and are capable of antigen presentation to naive T cells" [4]. Due to their ability to differentiate into myofibroblasts in culture, fibrocytes are implicated in the fibrogenesis of skin, lungs, kidneys, and liver [5,6]. In addition to collagen Type I, fibronectin and vimentin, fibrocytes express CD45, CD34, MHCII, MHCI, CD11b, Gr-1, and secrete growth factors (transforming growth factor (TGF)-β1, monocyte chemotactic factor (MCP)-1) that promote deposition of extracelluar matrix proteins [7]. Upon injury or stress, fibrocytes proliferate and migrate to the injured organ [5,7,8]. The number of recruited fibrocytes has been reported to vary from 25% in lung fibrosis [9,10] to ≈3-5% in liver fibrosis (e.g. BDL and CCl_4_) [11] of the collagen expressing cells, suggesting that the magnitude of fibrocyte differentiation into myofibroblasts depends on the organ and the type of injury. Interestingly, human serum amyloid protein (hSAP), which inhibits the differentiation of monocytes into fibrocytes, has been shown to inhibit fibrosis in lungs, kidneys and the liver [12-14]. This suggests that fibrocytes may have a role in fibrosis that is greater than their quantitative contribution to the myofibroblast population. In particular, fibrocytes support innate and adaptive immune responses [15].

To assess the potential contribution of fibrocytes in liver fibrosis, the Coll-GFP mice were used as donors in bone marrow transplantation into wild-type recipient mice. After recovery from the transplantation, these mice underwent live injury by CCl4 or BDL. In this way any GFP+ (i.e. collagen Type I expressing) cell found in the liver had to be derived from the bone marrow. This study demonstrated the approximately 5% of collagen Type I expressing cells in the injured liver were from the bone marrow. In particular, these cells fulfilled the definition of the fibrocytes in that they were GFP+ (expressing collagen Type I) and CD45+. Additional immunohistochemistry studies confirmed that these cells were expressing the collage Type I protein [11,16].

Next, the role of EMT was assessed using cell type specific CRE transgenic mice crossed with floxed ROSA26 beta galactosidase reporter gene mice. To assess the role of hepatocytes undergoing EMT into myofibroblasts, the albumin CRE reporter mice were used. Interestingly, when primarily cultures of hepatocytes from these transgenic mice were incubated with TGFbeta1, the hepatocytes changed their confirmation to appear more fibroblast like and started expressing the collagen Type I. Therefore, it appeared that hepatocytes can undergo EMT in primary cultures. However, when these reporter mice underwent CCl4 induced liver fibrosis, the results were completely different. *In vivo*, there was strong expression of Coll-GFP reporter in pericentral zones of the liver with bridging fibrosis as expected. However, none of the GFP+ myofibroblasts also expressed beta galactosidase [17]. Purification of the non-parenchymal cells from the injured liver confirmed that none of the collagen type 1 expressing cells were previously hepatocytes i.e. were expressing the ROSA26 beta galactosidase reporter. Thus, *in vivo*, there is no evidence that collagen producing cells (myofibroblasts) originate from hepatocytes in CCl4 induced liver fibrosis in mice [17].

Assessing the potential role of cholangiocytes to undergo EMT required an inducible CK19 CRE transgenic mouse. This is because CK19 is expressed in many epithelial cells during development, but only in cholangiocytes in the liver in the adult mouse. As predicted, the inducible CK19 specifically was expressed in cholangiocytes in the liver in the adult mouse. When compared to markers of myofibroblasts, there was no overlap between the expression of the CK19 YFP reporter and immune-fluorescence for alpha smooth muscle actin, FSP1, or desmin [18].

FSP1 (also called S100A4) is expressed in fibroblasts and has been proposed as a marker of EMT. FSP1 is strongly expressed in the injured liver. Surprisingly, it does not co-expressed in cells with markers of the mesenchymal phenotype, such as type 1 collagen or alpha smooth muscle actin. Therefore, we investigated which cells were actually expressing FSP1 in the injured liver. In culture, FSP1 was strongly expressed in fibroblasts derived from mouse ears as expected. However, it was not expressed in activated hepatic stellate cells in culture. Instead, the FSP1+ cells from the injured liver purified in the Kuffer cells/macrophage fraction of the non-parenchymal hepatic cells. By fluorescent sorting for FSP1+ cells using an FSP1-GFP reporter mouse, we were able to purify to homogeneity these FSP1+ cells. These cells then underwent gene expression microarray analysis and then compared to all available gene expression microarrays. An ontology analysis revealed that these FSP1+ cells were closest in resembling activated macrophage and bone marrow derived dendritic cells. A subsequent ontology analysis showed that FSP1+ cells closely resembled activated macrophage [19]. The function of these cells in the injured liver is unknown.

Fibroblasts are primarily located in the portal tract in the normal liver. Recent studies [20] had demonstrated that thy-1 is a potential marker of activated myofibroblasts in the injured liver. This study demonstrated an overlap in experimental fibrosis between thy-1 and alpha smooth muscle actin, implying that some myofibroblasts are derived from fibroblasts in liver fibrosis. Studies from other researchers have proposed that TE-7, an antibody against elastin, specifically identifies fibroblasts in the liver [21,22].

Several markers have been proposed to be specific for hepatic stellate cells, whether in the quiescent or activated state. These include the florescence of Vitamin A in the lipid droplets, GFAP, p75 NGF receptor, and synaptophysin [23-26]. Using these markers one should be able to distinguish between myofibroblasts that originate from fibroblasts or from hepatic stellate cells in experimental liver fibrosis.

From our studies and the published studies from other laboratories, we have concluded that in experimental models of liver fibrosis, most fibrogentic cells (myofibroblasts) are endogenous to the liver. It appears that the activated hepatic stellate cells and fibroblasts are the major endogenous fibrogenic cells, and that now these two cell types can be distinguished *in vivo*. On the other hand, fibrocytes migrate from the bone marrow to the liver where they contribute to inflammation and fibrosis, but are a minor component of the liver myofibroblast population. In some model systems and in patients with primary sclerosing cholangitis, there is evidence for EMT of the injured cholagiocytes on the basis of the co-expression of mesenchymal and epithelial markers, but no one has reported any clear evidence of epithelial cells becoming myofibroblasts. FSP1 (S100A4) identifies a myelomonocytic cell and not a fibroblast or myofibroblast in fibrotic mouse liver. Using genetic cell fate mapping, neither cholangiocytes nor hepatocytes transform into fibrogentic cells (myofibroblasts) in mouse models of liver fibrosis.

## Competing interests

The authors declare that they have no competing interests.
